# How do introgression events shape the partitioning of diversity among breeds: a case study in sheep

**DOI:** 10.1186/s12711-015-0131-7

**Published:** 2015-06-17

**Authors:** Grégoire Leroy, Coralie Danchin-Burge, Isabelle Palhière, Magali SanCristobal, Yann Nédélec, Etienne Verrier, Xavier Rognon

**Affiliations:** AgroParisTech, UMR1313 Génétique Animale et Biologie Intégrative, F-75231 Paris 05, France; INRA, UMR1313 Génétique Animale et Biologie Intégrative, F-78352 Jouy-en-Josas, France; Institut de l’Élevage, F-75595 Paris 12, France; INRA, UMR1388 Génétique, Physiologie et Systèmes d’Élevage, F-31326 Castanet-Tolosan, France; Université de Toulouse INPT ENVT, UMR1388 Génétique, Physiologie et Systèmes d’Élevage, F-31076 Toulouse, France; Université de Toulouse INPT ENSAT, UMR1388 Génétique, Physiologie et Systèmes d’Élevage, F-31326 Castanet-Tolosan, France

## Abstract

**Background:**

From domestication to the current pattern of differentiation, domestic species have been influenced by reticulate evolution with multiple events of migration, introgression, and isolation; this has resulted in a very large number of breeds. In order to manage these breeds and their genetic diversity, one must know the current genetic structure of the populations and the relationships among these. This paper presents the results of a genetic diversity analysis on an almost exhaustive sample of the sheep breeds reared in France. Molecular characterization was performed with a set of 21 microsatellite markers on a collection of 49 breeds that include five breed types: meat, hardy meat, dairy, high prolificacy and patrimonial breeds.

**Results:**

Values of expected heterozygosity ranged from 0.48 to 0.76 depending on the breed, with specialized meat breeds exhibiting the lowest values. Neighbor-Net, multidimensional analysis or clustering approaches revealed a clear differentiation of the meat breeds compared to the other breed types. Moreover, the group that clustered meat breeds included all the breeds that originated from the United Kingdom (UK) and those that originated from crossbreeding between UK breeds and French local breeds. We also highlighted old genetic introgression events that were related to the diffusion of Merino rams to improve wool production. As a result of these introgression events, especially that regarding the UK breeds, the breeds that were clustered in the ‘meat type cluster’ exhibited the lowest contribution to total diversity. That means that similar allelic combinations could be observed in different breeds of this group.

**Conclusions:**

The genetic differentiation pattern of the sheep breeds reared in France results from a combination of factors, i.e. geographical origin, historic gene flow, and breed use. The Merino influence is weaker than that of UK breeds, which is consistent with how sheep use changed radically at the end of 19^th^ century when wool-producing animals (Merino-like) were replaced by meat-producing breeds. These results are highly relevant to monitor and manage the genetic diversity of sheep and can be used to set priorities in conservation programs when needed.

**Electronic supplementary material:**

The online version of this article (doi:10.1186/s12711-015-0131-7) contains supplementary material, which is available to authorized users.

## Background

It is generally accepted that sheep domestication occurred about 11 000 years ago in a region of the Middle East along the Taurus-Zagros arc, probably through several domestication events [1, 2]. Since then, domesticated sheep have spread throughout the world, following human migration roads, and have been selected for different purposes and environments [2, 3]. As a consequence of reticulated evolution (i.e. multiple episodes of migration, introgression, and isolation), the current genetic structure of domesticated sheep is rather complex [4] with 1129 breeds reported by the FAO [5]. With 57 sheep breeds officially recognized, France is an interesting example on how sheep populations are genetically structured. Until the 19^th^ century, local sheep populations were differentiated according to their regions of origin (mainly, the northern part of France, and the Alps, Pyrénées and Massif Central mountains) [6]. Since the end of the 18^th^ century, several introgression events have been very well documented. The first major event was the use of Merino rams from a national flock that is still maintained at the Bergerie Nationale of Rambouillet. It was imported from Spain in 1786 and promoted by the Napoleonian administration to improve the French breeds’ wool quality (e.g. [7]). Then, because at the national level wool demands decreased from the 1860s [8], sheep breeding aimed at improving meat production by two successive episodes of sheep importation from United Kingdom. The first rams imported belonged to the “Longwool” group while the breeds imported in the second episode belonged to the “Down” group. At the end of the 19^th^ century, flock-books were created and since then, selection programs have been implemented in the sheep populations that had been impacted by those introgression events at different levels.

Several studies have investigated the genetic structure of sheep breeds based on molecular markers [4, 9–13] and showed that sheep breed differentiation depends on geographical origin, Merino introgression and/or breed use. Molecular tools are also useful to investigate conservation issues and the contribution of various populations to genetic diversity at different scales [14, 15]. However, although there are many sheep breeds in France, few molecular-based studies have been carried out to analyze their diversity.

Our aim was to investigate the genetic structure of sheep breeds in France using a near complete sample of the populations that are maintained over the country. For this purpose, 1826 individuals from 49 breeds were genotyped using 21 microsatellite markers, which allowed us to assess the genetic diversity of sheep breeds in France in relation to their history and conservation policy issues.

## Methods

### Sample collection and genotypes

Fifty-one populations belonging to 49 sheep breeds raised in France were sampled (Table 1). These populations belonged to five breed types (Table 1): (i) 15 meat breeds (M), (ii) five dairy breeds (D) among which, the Lacaune dairy breed comprised two subpopulations, (iii) three high prolificacy breeds (P), (iv) 25 hardy meat breeds (H) among which, the Lacaune meat breed comprised two subpopulations, and (v) one patrimonial breed (Pa), i.e. the Mérinos de Rambouillet breed (MeRa). For the sake of clarity, the four subpopulations of the Lacaune breed will be considered hereafter as separate breeds. The geographical coordinates (latitude and longitude) relative to the region of origin were determined for each breed. A total of 1826 individuals were sampled and the number of animals per breed ranged from 12 (Romanov) to 55 (Roussin de la Hague). When pedigree data were available, animals that were as little related as possible were chosen. For the six breeds (BeIl, Land, LaBr, MoNo, RoRo and ThMa) for which there was no pedigree data, animals were sampled from as many different birth flocks and birth periods as possible.

**Table 1:** Name, sample size and region of origin for the 48 sheep breeds

Breed code	Breed name (in French)	Number	Type^a^	Status^b^	Region of origin^d^		
					Geographical region^c^	Latitude	Longitude
AuCa	Aure et Campan	34	H	2	MC	42.9	0.4
Avra	Avranchin	26	M	2	NW	48.7	−1.4
BaBe	Basco-Béarnaise	36	D	1	SW	43.1	−0.4
Bare	Barégeoise	19	H	2	SW	42.9	0.1
BeCh	Berrichon du Cher	40	M	1	Pl	47.1	2.4
BeIl	Belle-Île	36	P	3	Pl	46.6	2.0
BeIn	Berrichon de l’Indre	40	H	3	Pl	47.3	−3.2
Bize	Bizet	28	H	2	MC	45.3	3.4
BlMa	Bleu du Maine	42	M	2	NW	48.0	−0.5
BlMC	Blanc du Massif Central	40	H	1	MC	44.5	3.5
Boul	Boulonnaise	40	H	2	Pl	50.3	2.8
CaLo	Causses du Lot	40	H	1	MC	44.8	1.6
Cast	Castillonnaise	26	H	3	SW	42.9	1.0
Char	Charmoise	30	M	1	Pl	47.4	1.3
Cors	Corse	27	D	1	SE	42.1	9.5
Cote	Cotentin	35	M	2	NW	49.0	−1.3
DoDo	Dorset-Down	20	M	2	UK	50.7	−2.3
EsLM	Est à Laine Mérinos	40	H	2	Pl	48.7	6.2
Griv	Grivette	38	H	2	SE	45.2	5.7
Hamp	Hampshire	39	M	2	UK	51.1	−1.3
IlFr	Ile de France	34	M	1	Pl	48.8	2.4
Lacaune (*4 subpopulations*)					MC	44.0	3.0
LaLC	Lacaune Lait - Confédération	40	D	1			
LaLO	Lacaune Lait - OviTest	40	D	1			
LaVG	Lacaune Viande - Gebro	40	H	1			
LaVO	Lacaune Viande - OviTest	40	H	1			
Land	Landaise	29	H	3	SW	44.1	−0.7
LaBr	Landes de Bretagne	30	H	3	NW	47.4	−2.1
Limo	Limousine	34	H	2	MC	45.6	2.1
Lour	Lourdaise	34	H	3	SW	43.1	0.0
MaTN	Manech Tête Noire	34	D	1	SW	43.1	−1.4
MaTR	Manech Tête Rousse	40	D	1	SW	43.3	−1.4
MeAr	Mérinos d’Arles	40	H	2	SE	43.6	4.8
MeRa	Mérinos de Rambouillet	40	Pa	3	Pl	48.6	1.8
MoCh	Mouton Charollais	40	M	1	Pl	46.4	4.3
MoNo	Montagne Noire	37	H	2	MC	43.5	2.4
Mour	Mourerous	35	H	2	SE	44.1	6.9
MoVe	Mouton Vendéen	43	M	1	NW	46.5	−0.8
NoVe	Noire du Velay	45	H	2	MC	45.0	3.8
PrSu	Préalpes du Sud	40	H	2	SE	44.2	5.9
Rava	Rava	39	H	2	MC	45.8	3.1
RoHa	Roussin de la Hague	55	M	2	NW	49.6	−1.8
Roma	Romane	38	P	1	Pl	46.4	0.9
RoOu	Rouge de l’Ouest	47	M	1	NW	47.5	−0.6
Roov	Romanov	12	P	2	Ru	55.0	7.0
RoRo	Rouge du Roussillon	30	H	3	SW	43.0	2.6
Solo	Solognote	35	H	3	Pl	47.6	2.0
Sout	Southdown	20	M	2	UK	51.4	−2.4
Suff	Suffolk	41	M	1	UK	52.2	1.0
Tara	Tarasconnaise	32	H	2	SW	42.8	1.6
Texe	Texel	47	M	1	Ne	53.1	4.8
ThMa	Thones et Marthod	39	H	3	SE	45.9	6.3

^a^H = hardy meat breed; M = meat breed; P = high-prolificacy breed; D = dairy breed; Pa = patrimonial breed; ^*b*^1 = mainstream breeds; 2 = local breeds (not rare); 3 = rare breeds; ^***c***^MC = Massif Central; Ne = Netherlands; NW = North-West; Pl = Plain; Ru = Russia; SE = South-East; SW = South-West; UK = United-Kingdom; ^***d***^geographical coordinates (latitude and longitude): coordinates of the region of origin for each breed

Twenty one microsatellite markers were used to perform the analysis. Eight of these microsatellites came from the French panel for parentage testing (*CSRD0247, HSC, INRA49, McM42, MAF65, McM527, MAF0214, OaRFCB20*). The 13 other microsatellites (*HUJ616, ILSTS005, ILST011, MAF209, MAF70, OarFCB128, OaRCP34, OaRFCB193, OaRFCB304, OaRJMP29, OarJMP58, SR-CRSP9, BM8125*) were chosen from those available in the UE Econogen project [16]. Thirteen of the 21 selected loci were part of the ISAG-FAO recommended microsatellite markers. Amplifications and analyses of all the samples were performed by the same laboratory (Labogena, France), using a capillary sequencer (ABI PRISM 3100 Genetic Analyzer, Applied Biosystems).

### Statistical analysis

The presence of null alleles was tested using FreeNA [17] i.e. loci with an estimated frequency of null alleles (*r*) higher than 0.2 were considered as potentially problematic for calculations [9]. Allele frequencies, numbers of alleles, observed heterozygosity (*Ho*), non-biased expected heterozygosity (*He*), effective number of alleles (*Ae*) and F-statistics [18] were estimated with GENETIX *4.05.2* [19]. GENEPOP *4.07* [20] was used to evaluate departure from Hardy-Weinberg equilibrium and pairwise genic differentiation among breeds [21]. Allelic richness (*Ar*) was computed with the rarefaction method using FSTAT *2.9.3.2* [22]. Significance levels of the tests were corrected with sequential Bonferroni correction on loci. Potential hierarchical genetic structure was investigated with the AMOVA procedure implemented in ARLEQUIN *3.5.1.2* [23]. Breeds were divided into different groups according to: (i) type (dairy, meat, hardy meat, and high prolificacy; see Table 1) or (ii) original geographic location (Massif Central, South-West, South-East, North-West, Plain from Center/Northern part of France, and the United-Kingdom (UK); see Table 1). The Mérinos de Rambouillet breed (MeRa) was excluded from all the AMOVA analyses because this breed is the unique representative of its type (patrimonial). Romanov (Roov) and Texel (Texe) breeds were also excluded because they were the only representatives of their geographical groups (respectively, Russia and The Netherlands). Significance levels were determined after 16 000 permutations.

The matrix of Reynolds distances (*D*_*R*_ [24]) was computed using PHYLIP 3.69 [25] and used to draw a Neighbor-Net [26] network with SPLITSTREE *4.5* [27]. A principal component analysis was also performed using PCAGEN [28]. The significance of the axis was evaluated using permutation tests (1000 randomizations of the genotypes).

Clustering approaches were performed on the 51 breeds using a Bayesian clustering procedure implemented in STRUCTURE [29] with the number of *K* clusters ranging from 1 to 10 and then equal to 15, 20, 25, 30, 35, 40, 45, 48, 51, and 55. For each value of *K*, 50 runs were performed with 1 000 000 iterations following a burn-in period of 100 000, under the admixture and correlated allele frequency model. Since consistency across runs seems to be an informative method for assessing species structure across breeds [30, 31], we used CLUMPP [32] to estimate the similarity function G’ over runs for the different values of *K*, using the LARGEKGREEDY algorithm. We selected a subset of runs that included the run with the highest number of similar runs (symmetric similarity coefficients (SSC) greater than 0.90) grouped with the corresponding similar runs. We used this subset to compute a mean **Q**-matrix. Breed assignment was performed as in Leroy et al. [31]. Animals were considered as correctly assigned to their breed if they were primarily associated to the cluster that included the largest number of animals belonging to the breed, using results for *K* = 51. For clusters that comprised two breeds, runs were performed for *K* = 2 using only the breeds that were associated within the sub-cluster.

The contribution of each breed to the diversity of the whole set of breeds was computed according to the method of Caballero and Toro [33]. Let *p*_*ki*_ be the average frequency of allele *k* in breed *i*, then, the average coancestry between breeds *i* and *j* is:

1 $$ {f}_{ij}={\varSigma}_k\kern0.5em {p}_{ki}{p}_{kj} $$

When several markers are used, coancestry is averaged over loci. The total genetic diversity (*GD*_*T*_) is assumed to be the sum of the within-breed genetic diversity (*GD*_*WS*_) and the between-breed genetic diversity (*GD*_*BS*_):

2 $$ G{D}_T= 1\mathit{\hbox{-}}{\varSigma}_i\kern0.5em {\varSigma}_j\kern0.5em {f}_{ij}/{n}^2\kern0.5em , $$

3 $$ G{D}_{WS}= 1-{\varSigma}_i\kern0.5em {f}_{ii}/n, $$

4 $$ G{D}_{BS}={\varSigma}_i\kern0.5em {\varSigma}_i\kern0.5em {D}_{ij}/{n}^2 $$

In these equations, *n* is the number of breeds and *D*_*ij*_ is Nei’s minimum distance between breeds *i* and *j*. Contribution of a breed to the diversity of the whole set of breeds was computed by the loss or gain of diversity *∆GD* when the breed is removed.

## Results

### Genetic diversity

For the complete dataset of breeds and markers, 357 alleles were identified. The average number of alleles per locus was 17 and ranged from nine (loci *OarCP3* and *ILSTS011*) to 28 (locus *UHJ616*).

Heterozygosities, mean number of alleles (*MNA*), effective number of alleles (*Ae*) and allelic richness (*Ar*) are in Table 2. *He* ranged from 0.48 in the Belle-Île breed (BeIl) to 0.76 in the *Corse* breed (Cors), with a mean value of 0.66 (±0.07). *He* were significantly higher in hardy meat breeds (*P* < 0.0001; Wilcoxon-Mann Whitney test) and dairy breeds (*P* = 0.0005) than in specialized meat breeds, whereas differentiation between hardy meat and dairy breeds was not significant (*P* = 0.68). *Ae* ranged from 2.14 (BeIl) to 5.18 (Cors), with a mean value of 3.64 (±0.72). *Ar* values (computed for breeds with at least 18 individuals genotyped for each locus) varied from 2.96 (MeRa) to 7.72 (Cors), with a mean value of 5.6 (±1.05). *F*_*IS*_ per breed ranged from −0.058 (Rava) to 0.117 (LaBr). The larger *He*, *Ae* and *Ar* values obtained for the Cors breed are probably related to a lower selection intensity than for other dairy breeds, linked to an extensive production system. After sequential Bonferroni correction, five breeds showed a significant deficit of heterozygotes for one locus, and one breed carried one locus with an excess of heterozygotes. Only one locus per breed combination out of 1071 was identified with a potential null allele (*r* > 0.2; data not shown) i.e. the *McM42* locus in the LaBr breed. However, excluding this locus had very minor effects on *F*_*IS*_ and *He* (Wilcoxon test; *P*-value > 0.05), which suggests that null alleles are not the main cause of significant *F*_*IS*_ values (data not shown). Thus, we chose to conserve all 21 loci. Implementation of the pair-wise population differentiation test in the software GENEPOP 4.07 [20] showed that all breed pairs were significantly differentiated, including the six breed pairs that included the four Lacaune subpopulations. *F*_*IS*_, *F*_*IT*_ and *F*_*ST*_ values were equal to −0.001, 0.12 and 0.12, respectively.

**Table 2:** Summary of genetic diversity measures across the 51 populations

Breed code	He	Ho	MNA	Ae	Ar (N ≥ 18)	F_IS_	Excess HWE	Deficiency HWE	Assignement (%) (Bayesian clustering)
AuCa	0.70	0.72	7.81	4.31	6.72	−0.019			91.2
Avra	0.52	0.54	4.57	2.72	4.28	−0.035			100
BaBe	0.71	0.72	7.05	3.98	6.15	−0.012			100
Bare	0.72	0.71	7.43	4.77	-	0.015			-^a^
BeCh	0.55	0.57	4.91	2.84	4.45	−0.038			97.5
BeIl	0.48	0.50	3.62	2.14	3.40	−0.042			100
BeIn	0.65	0.65	6.19	3.58	5.42	0.001			100
Bize	0.65	0.64	5.81	3.62	5.36	0.024			100
BlMa	0.56	0.56	5.24	2.76	4.53	0.003		1	90.5^f^
BlMC	0.72	0.73	8.14	4.45	-	−0.017			62.5
Boul	0.65	0.65	5.48	3.51	5.02	−0.006			97.5
CaLo	0.62	0.60	5.86	3.30	5.25	0.029			100
Cast	0.71	0.71	6.24	4.38	5.99	0.008			76.9
Char	0.54	0.55	4.62	2.49	4.25	−0.019			100
Cors	0.76	0.75	8.48	5.18	7.72	0.013			77.8
Cote	0.53	0.53	4.29	2.56	3.93	−0.016		1	100
DoDo	0.67	0.63	6.10	3.71	5.99	0.060			60
EsLM	0.71	0.73	6.95	4.20	6.14	−0.024			95
Griv	0.71	0.71	7.76	4.23	6.63	0.003			100
Hamp	0.67	0.66	6.05	3.59	5.55	0.016			94.9^e^
IlFr	0.66	0.66	5.52	3.60	5.10	−0.006			100
LaBr	0.55	0.49	4.71	2.43	4.26	0.117**		1	96.7^b^
LaLC	0.69	0.68	7.00	3.90	6.02	0.020			50
LaLO	0.67	0.69	6.71	3.66	5.82	−0.031			77.5
LaVG	0.69	0.69	6.67	3.87	5.75	−0.001			92.5
LaVO	0.71	0.72	7.91	4.25	6.79	−0.021			65
Land	0.68	0.69	5.57	3.44	5.10	−0.014			93.1
Limo	0.72	0.72	7.33	4.45	6.44	−0.014		1	91.2
Lour	0.71	0.74	7.24	4.48	6.48	−0.039			76.5
MaTN	0.71	0.71	6.91	4.31	6.23	0.004	1		100
MaTR	0.72	0.69	6.86	3.94	6.04	0.027			85
MeAr	0.74	0.75	8.10	4.38	6.90	−0.011			80
MeRa	0.50	0.50	3.05	2.32	2.96	0.005			100
MoCh	0.66	0.64	6.67	3.55	5.75	0.028			97.5^c^
MoNo	0.70	0.71	7.24	3.72	6.11	−0.010		1	78.4^b^
Mour	0.70	0.70	7.67	3.97	6.54	−0.004			74.3^b^
MoVe	0.61	0.60	6.33	3.14	5.49	0.010			100
NoVe	0.72	0.70	8.19	4.67	7.05	0.044			84.4
PrSu	0.71	0.68	7.81	4.13	6.61	0.034			90
Rava	0.69	0.72	6.95	3.89	6.15	−0.058*			89.7
RoHa	0.58	0.58	5.71	2.72	4.76	−0.010			98.2
Roma	0.70	0.71	6.95	3.98	6.21	−0.002			100^d^
RoOu	0.61	0.60	6.38	3.20	5.35	0.018			95.7^f^
Roov	0.61	0.61	4.33	3.01	-	0.005			100^d^
RoRo	0.72	0.76	7.71	4.35	6.89	−0.055			86.7
Solo	0.60	0.60	4.62	2.91	4.20	−0.012			100
Sout	0.57	0.59	4.38	2.66	4.30	−0.030			100^c^
Suff	0.61	0.59	5.14	3.13	4.55	0.027			100^e^
Tara	0.71	0.70	8.05	4.02	7.04	0.016			46.9
Texe	0.65	0.66	5.62	3.14	4.86	−0.012			100
ThMa	0.69	0.68	7.10	4.18	6.06	0.014			97.4

*He* = non-biased expected heterozygosity; *Ho* = observed heterozygosity; *MNA* = mean number of alleles per locus; *Ae* = effective number of alleles per locus; *Ar* (N **≥** 18) = allelic richness computed for populations with more than 18 individuals genotyped for each locus; *F*_IS_* = significant value after sequential Bonferroni correction (test significance: * *P* < 0.05. ** *P* < 0.01); Deficiency HWE/Excess HWE = number of Loci in Heterozygote Deficiency/Excess, after sequential Bonferroni correction; ^a^never more than two individuals clustered together; ^b^the breed is associated with two different private clusters (see text); ^c,d,e,f^results are provided considering the cluster shared by the two breeds for K = 51; these two breeds were however clearly segregated when analyzed separately (see text)

### Breed relationships and clustering

**Fig. 1:**
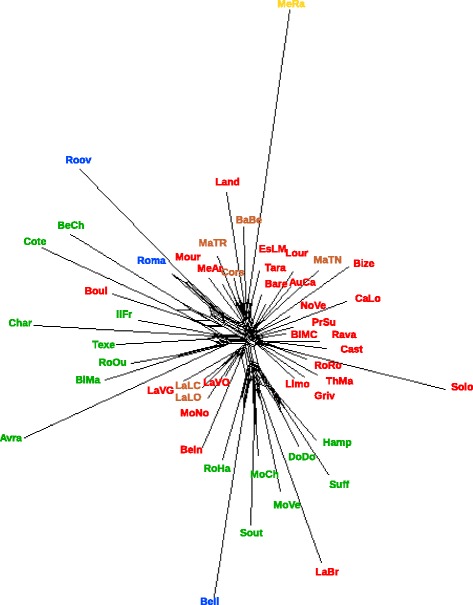
Neighbor-Net network for the 51 sheep populations, based on Reynold’s *D*_*R*_ distance. Brown = dairy breeds; red = hardy meat breeds; yellow = patrimonial breed; green = meat breeds and blue = high-prolificacy breeds

The Neighbor-Net network based on *D*_*R*_ distance (Fig. 1) formed a star-like pattern, with several clusters. Meat breeds (M) were clustered within two groups that included a few other breeds. Notably, one group included the four meat breeds that originated from the United Kingdom (Suff, Hamp, DoDo and Sout), three French meat breeds (RoHa, MoCh and MoVe), one high prolificacy breed (BeIl) and one hardy meat breed (LaBr). The other group clustered seven French meat breeds (Avra, BlMa, RoOu, Char, IlFr, Cote and BeCh), one breed from The Netherlands (Texe), one hardy meat breed (Boul) and the two other high prolificacy breeds (Roma and Roov).

**Fig. 2:**
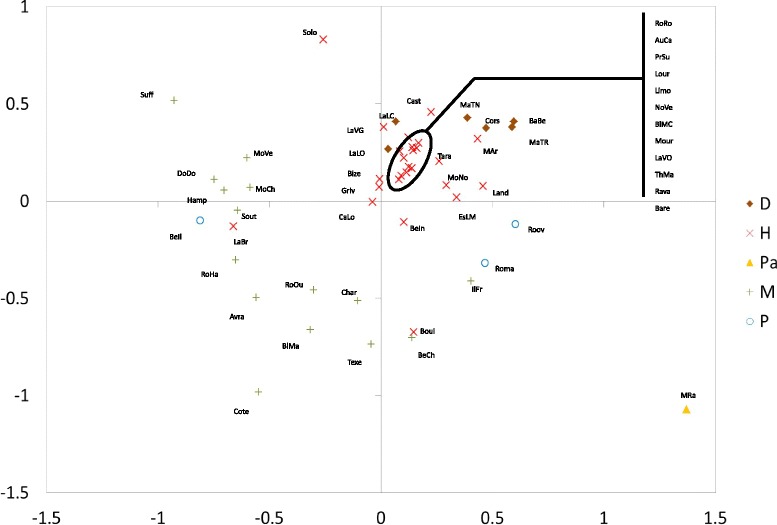
Principal component analysis for the 51 sheep populations. The projection is shown on the first two axes. Population codes are in Table 1. Brown diamonds = dairy breeds; red crosses = hardy meat breeds; yellow triangles = patrimonial breed; green plus signs = meat breeds; blue circles = high-prolificacy breeds. Axis 1: 9.9 % inertia; *P*-value = 0.001. Axis 2: 8.6 % inertia; *P*-value = 0.001

PCA analysis (Fig. 2) displayed a clear differentiation between the meat breeds and the hardy meat and dairy breeds. All meat breeds except BeCh and IlFr were plotted on the left side of the figure, whereas all dairy and hardy meat breeds (except five) clustered within the bottom right quadrant. Only two hardy meat breeds Boul and LaBr were plotted very close to the meat breeds. One high prolificacy breed (BeIl) was plotted close to the meat breeds, whereas the two other high prolificacy breeds (Roma and Roov) were on the other side (top right quadrant) near the BeCh and IlFr breeds. The patrimonial Mérinos de Rambouillet breed (MeRa), was clearly isolated from all other breeds, probably because of its low level of genetic variability i.e. this population has been maintained as a closed flock since around 230 years).

**Fig. 3:**
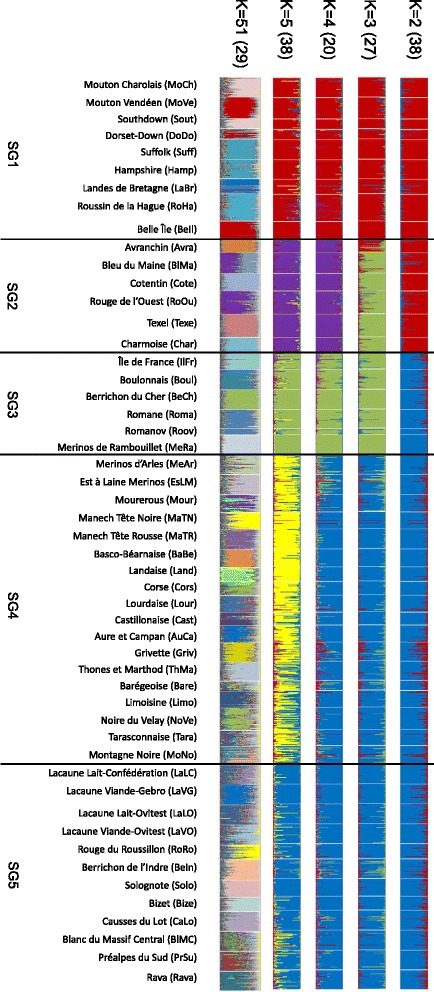
Estimated membership coefficients of each individual in the inferred *K* cluster, with *K* = 2 to 5 and *K* = 51. In brackets, number of runs with similar solutions (SSC > 0.90) that was used to compute the mean Q-matrix

**Fig. 4:**
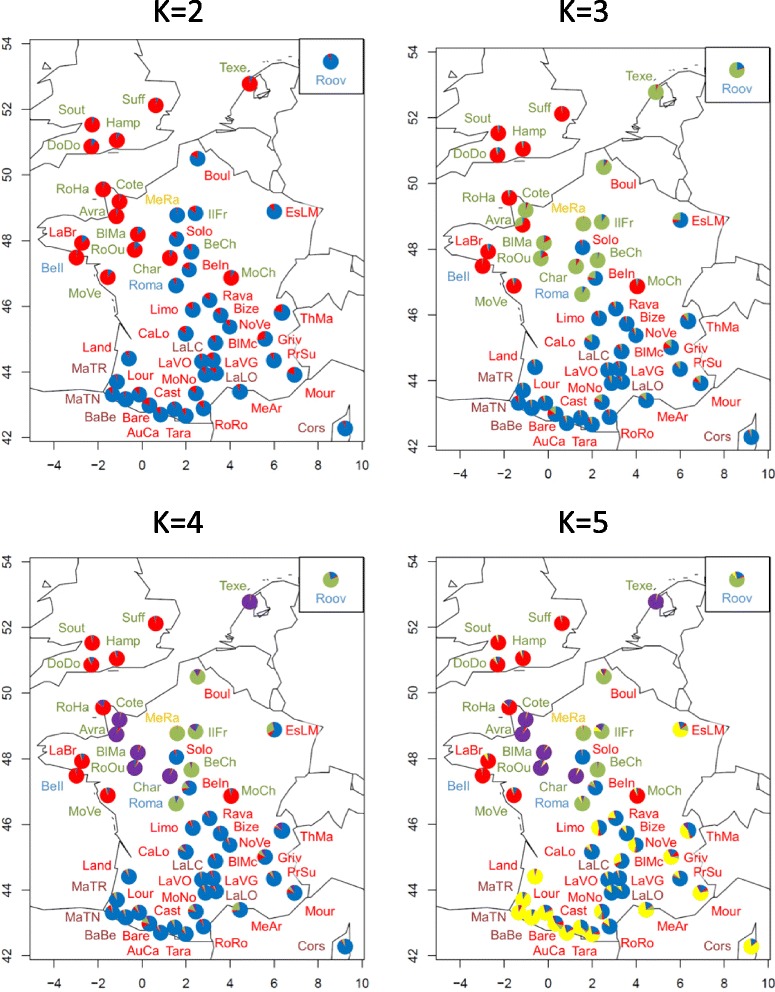
Geographical interpolation of structure results for *K* = 2 to 5 using the mean Q-matrix over runs with similar solutions. Breeds are distributed according to their location of origin. Brown = dairy breeds; red = hardy meat breeds; yellow = patrimonial breed; green = meat breeds; blue = high-prolificacy breeds. Each pie shows for a given breed the proportions of membership coefficients relative to clusters (see Fig. 3)

Bayesian clustering methods provided complementary information on the genetic relationships among the populations. For these, we used the **Q**-matrix averaged over the most similar runs (Fig. 3) for *K* = 2 to 5 and 51 (or overall runs for *K* = 2 to 10 and 51 [See Additional file 1: Figure S1]) and a combined analysis of the distribution of membership coefficients according to breed and geographical location (location of origin) of these breeds (Fig. 4; *K* = 2 to 5). Likelihood values (Ln(P(D))) across runs reached a plateau when *K* was close to 45 [See Additional file 2: Figure S2]. With *K* = 2, a group that comprised all the breeds from the UK (South, DoDo, Suff, and Hamp), the Netherlands (Texe) and nine western French breeds (MoVe, LaBr, RoHa, BeIl, Avra, BlMa, Cote, RoOu, and Char) and the Mouton Charollais (MoCh) breed was clearly differentiated from a second group that included all the other breeds. As *K* increased, this first group segregated in two subgroups, one including the UK breeds and MoCh, MoVe, LaBr, RoHa, and BeIl breeds (i.e. SubGroup 1 or *SG1*) and the other including Texe, Avra, Cote, RoOu, and Char breeds (*SG2*).

From *K* = 3 to 4, a third subgroup (*SG3*) that included IlFr, Boul, BeCh, Roma, Roov, and MeRa breeds was separated from the second group. As *K* increased (3 to 10), the breeds in *SG3* clustered together. However the Mérinos de Rambouillet breed (MeRa) and the Île-de-France breed (IlFr) separated at *K* = 7 and *K* = 10, respectively [See Additional file 1: Figure S1]. For the other breeds, two subdivisions occurred as *K* increased to 5 i.e. *SG4* and *SG5. SG4* included the MeAr, EsLM, and Mour breeds, the Southwestern breeds (MnTN, MnTR, BaBe, Land, Lour, Cast, AuCa, Bare, Limo, and Tara), two Alpine breeds (Griv and ThMa), one Mediterranean breed (Cors) and two breeds from the Massif Central (NoVe and MoNo; although the MoNo breed is now bred in the Southwest area). The last subdivision (*SG5*) included all the other breeds i.e. from the Massif Central, except for the Préalpes du Sud breed (PrSu, in the Alps) with a differentiation pattern changing as *K* increased.

For *K* = 51, 41 breeds were assigned to a private cluster, i.e. they were primarily associated to the cluster that clustered the largest number of animals that belonged to the breed. For the three breeds LaBr, MoNo, and Mour, each one was associated with two clusters that consisted mainly of individuals that belonged to the same breed, and these pairs of clusters were considered as private breed clusters. Four pairs of breeds shared the same cluster BlMa/RoOu, Hamp/Suff, MoCh/Sout, and Roma/Roov, respectively. Each pair of breeds was analyzed individually (data not shown) and, in each case, individuals of the two breeds were assigned in their own private cluster. In contrast, one breed, the Barégeoise breed (Bare), was not assigned to a specific cluster and all Bare individuals except two pairs were assigned to different clusters. Finally, excluding the Barégeoise breed, 90.1 % of the individuals of the 50 remaining breeds were assigned to their putative breed (Table 2), this percentage ranging from 47 % for the Tara breed to 100 % for 19 breeds.

### Partition of diversity

In the hierarchical analysis (Table 3), the “within-breed” component explained the largest part of the total genetic variance (88 to 89 %; *P* < 0.0001), regardless of the hypothetical breed structure tested. Two models of population structure i.e. breed types and geographical origin were investigated. The greatest variation among groups (1.55 % of the total variance; *P* < 0.0001) was observed with the geographical model compared to the breed type model (1.14 %; *P* < 0.0001).

**Table 3:** Hierarchical partitioning of the genetic variance (AMOVA)

Grouping^a^	Variance components (%)			F-statistics		
	Among groups	Among breeds within group	within breeds	*F*_*CT*_	*F*_*SC*_	*F*_*ST*_
All breeds in one group		11.01 ***	88.99 ***			0.110 ***
Breed types (*n* = 4)	1.14 ***	10.25 ***	88.60 ***	0.011 ***	0.104 ***	0.114 ***
Geographical areas (*n* = 6)	1.55 ***	9.69 ***	88.76 ***	0.016 ***	0.098 ***	0.112 ***

^a^ see text and Table 1; test significance: *** *P* < 0.0001

Contributions to the genetic diversity are in Table 4. Δ*GD*_*WS*_ ranged from −0.002 (Cors) to 0.0034 (BeIl), while Δ*GD*_*BS*_ ranged from −0.0038 (MeRa) to 0.0013 (Bare). The largest decrease in total gene diversity (Δ*GD*_*T*_) was observed when the *Corse* (Cors; − 0.0012), Landaise (Land; − 0.0012) or Basco-Béarnaise (BaBe; −0.0011) breeds were removed. In contrast, when the Roussin de La Hague (RoHa), Blanc du Massif Central (BlMa) or Belle-Île (BeIl) breeds were removed, diversity increased by 0.0016, 0.0014 or 0.0013, respectively.

**Table 4:** Contributions of the different breeds to genetic diversity, according to the method of Caballero and Toro [33]

Breeds	Δ*GD*_*WS*_ (x10^3^)	Δ*GD*_*BS*_ (x10^3^)	Δ*GD*_*T*_ (x10^3^)	*He*
Cors	−2.01	0.82	−1.19	0.76
Land	−0.48	−0.68	−1.16	0.68
BaBe	−1.04	−0.04	−1.07	0.71
Roov	1.24	−2.24	−0.99	0.61
MaTN	−1.11	0.16	−0.95	0.71
MeAr	−1.7	0.86	−0.83	0.74
MeRa	3	−3.82	−0.83	0.50
Roma	−0.99	0.26	−0.73	0.70
Lour	−1.16	0.51	−0.65	0.71
MaTR	−1.05	0.41	−0.64	0.71
Cast	−1.08	0.54	−0.54	0.71
EsLM	−1.22	0.75	−0.47	0.72
Boul	0.06	−0.43	−0.38	0.65
Griv	−1.1	0.73	−0.38	0.71
RoRo	−1.22	0.84	−0.37	0.72
Solo	1.21	−1.57	−0.36	0.59
Limo	−1.18	0.84	−0.35	0.72
Hamp	−0.27	−0.07	−0.34	0.67
Texe	−0.04	−0.28	−0.33	0.65
NoVe	−1.33	1.06	−0.27	0.72
MoNo	−0.95	0.68	−0.26	0.70
LaVG	−0.78	0.57	−0.21	0.69
Mour	−0.88	0.69	−0.19	0.70
AuCa	−0.96	0.78	−0.18	0.70
Bize	0.05	−0.22	−0.16	0.65
DoDo	−0.04	−0.06	−0.1	0.65
ThMa	−0.67	0.58	−0.1	0.69
BeIn	−0.03	−0.05	−0.08	0.66
MoCh	−0.15	0.09	−0.06	0.66
BlMc	−1.3	1.26	−0.04	0.72
IlFr	−0.12	0.09	−0.02	0.66
Prea	−1.08	1.13	0.05	0.71
Rava	−0.63	0.74	0.11	0.69
Bare	−1.13	1.27	0.15	0.72
LaVO	−1.06	1.21	0.15	0.71
LaLC	−0.8	0.97	0.17	0.69
Tara	−0.99	1.17	0.18	0.71
LaBr	2.12	−1.87	0.25	0.55
Suff	0.89	−0.56	0.33	0.61
MoVe	0.86	−0.51	0.35	0.61
LaLO	−0.39	0.85	0.46	0.67
Avra	2.62	−2.1	0.53	0.52
BeCh	2.1	−1.36	0.74	0.55
Sout	1.76	−0.92	0.84	0.57
CaLo	0.63	0.23	0.86	0.62
RoOu	0.82	0.06	0.88	0.61
Char	2.37	−1.48	0.89	0.54
Cote	2.54	−1.6	0.93	0.53
BeIl	3.42	−2.1	1.32	0.48
BlMa	1.77	−0.33	1.44	0.56
RoHa	1.47	0.14	1.61	0.58

Δ*GD*_*WS*_ = loss or gain of gene diversity within populations when the breed is removed; Δ*GD*_*BS*_ = loss or gain of gene diversity between populations when the breed is removed; Δ*GD*_*T*_ = loss or gain of total diversity when the breed is removed; *He*: non-biased expected heterozygosity

## Discussion

Investigating the genetic structure of sheep breeds that are raised in France, using an approach based on their geographical origin and not the regions where they are currently raised, provides interesting insights into the recent history of sheep breeding in France.

Results from the Bayesian approach clustered the breeds according to geographical origin and to the impact of the successive crossing events (Fig. 4). We showed that two groups *SG1* and *SG2* were influenced by genetic introgression from UK breeds. *SG1* includes breeds of French (BeIl, LaBr, MoCh, MoVe, RoHa) and UK origins (DoDo, Hamp, Suff, Sout) related to Down meat breeds (*SG1*). In the *SG2* group, for some breeds (Avra, BlMa, Char, Cote, RoOu), introgression of former UK Longwool breeds may still have a dominant influence on population clustering, since they cluster with the Texel breed, which has a Dutch origin and is also considered as genetically similar to the Longwool breeds [11]. Results for the breeds that cluster in the *SG3* group show that they are related to the extensive use of Merino rams at the end of 18^th^ and beginning of the 19^th^ century (“Merinization”) to improve wool production [6]. *SG3* includes Mérinos de Rambouillet (MeRa), which is the breed that was originally used for merinization in France, breeds (IlFr, Boul and BeCh) that were created by crossing MeRa with UK Longwool breeds such as the Dishley breed, and the Romane breed (Roma), which is a recent breed that was created by crossing the Berrichon-du-Cher (BeCh) and Romanov (Roov) breeds, Romanov being also included in the cluster. Three other breeds of Merino origin, i.e. the current Merino hardy meat breeds (MeAr, EsLM) and the Mourerous breed (Mour), are clustered in another group (*SG4*). All other breeds, including hardy meat or dairy breeds that originate from the south of France, are clustered according to two main geographical origins, namely South West (Pyrénées) of France for cluster *SG4* and Massif Central for *SG5*. Overall, the results from the Bayesian approach are consistent with what is known on the history of introgression events that took place in French sheep populations during the 19^th^ century [6], even if the Merino *SG3* group may appear artificial since it aggregates breeds that have been influenced by Merino and Romane (Roma) breeds (see above). Genetic drift and founder effects within the French Romanov breed (Roov) together with the small number of sampled Romanov individuals, may explain why this breed clustered within the *SG3* group. Neighbor-Net (Fig. 1) and PCA (Fig. 2) methods provided results that agree with the theoretical expectation since the Romane breed is placed between the two breeds that were crossed to create it. This is a good example to illustrate the need to combine different approaches when analyzing the genetic history of populations with molecular markers.

Independently from the breeds’ specific histories, our analysis also investigated to what extent breed type or geographical origin may account for genetic breed structure. The overall *F*_*ST*_ estimate (11.7 %) was consistent with that reported in previous studies on sheep breeds (~13 %; [9, 12]). Here, geographical origin and breed type explained 1.6 % and 1.1 %, respectively, of the total genetic variation, while Lawson-Handley et al. [9] found 1 % and 2.7 %, respectively in an analysis on European sheep breeds. Obviously, these two parameters are not independent from each other (Fig. 4). For instance, meat breeds, which mainly originate from the UK, were used to create the breeds from the northwestern part from France i.e. through strong introgression from Longwool or Down breeds. Specialization for meat types can be related to the socio-economic background of the northwestern part of France (combination of high demands for meat and availability of rich pastures) [6]. In contrast, dairy breeds are from three distinct origins, i.e. western Pyrénées (BaBe, MaTR, MaTN), Corsica (Cors) and the southern part of Massif central (LaLC, LaLO); these last two breeds have a different genetic background from the four previous breeds. Thus, based on these findings, it can be hypothesized that the genetic differentiation of sheep breeds in France results from a combination of geographical origin, historic gene flow, and breed use, in relation to the socio-economic background and the main farm systems that comprise specialized meat types and more intensive production in the northern part, and hardy (meat or dairy) types and more or less extensive farm systems in the southern part of the country [6].

Among the applications of this study for breed management, our results on breed assignment (90.1 % of individuals assigned to their putative breed) confirm that each breed constitutes a rather homogeneous genetic group. Even the four subpopulations from the Lacaune breed (LaLC, LaVG, LaLO, LaVO) that have been subjected to different selection programs for about 40 years, appeared relatively well differentiated (Fig. 3). Many of the detected misassignments were found for breeds with high levels of genetic diversity. For instance the Barégeoise breed (Bare), which is the breed with the third highest *He* (0.72), could not be assigned to any specific cluster. The relatively recent creation of this breed (beginning of the 20^th^ century) as a sub-population of the Lourdaise breed (Lour), with a flock-book that started only in 1975, and the former use of crossbreeding with several breeds [34] explains partly the high *He* as well as the high rate of misassignments. When the Barégeoise breed and the breeds that are considered as historically close (AuCa and Lour) or frequently used in crossbreeding (BeCh and Tara) were analyzed independently, they were each assigned to a private cluster, with Barégeoise individuals showing more heterogeneity (data not shown). Using a larger number of markers would probably improve the assignment results and allow to register individuals that lack a known pedigree within a given selection nucleus.

Regarding diversity partitioning, a high correlation was observed between *He* and various diversity components. This correlation was negative with Δ*GD*_*WS*_ (*r* = −0.99), positive with Δ*GD*_*BS*_ (*r* = 0.87), and negative with Δ*GD*_*T*_ (*r* = −0.62), which indicates that breeds with a high diversity level contribute more to total genetic diversity. It is interesting to note that the three breeds with the largest contribution to total diversity (Cors, Land and BaBe), i.e. with the most negative Δ*GD*_*T*_, showed a high level of heterozygosity, as expected, but also shared a similar genetic background according Neighbor-Net and Structure methods. Two of these breeds are local breeds (Cors and BaBe) and the other (Land) is a rare breed. The five breeds with the lowest contribution to total genetic diversity (i.e. the highest Δ*GD*_*T*_) also showed a low genetic diversity and were related either to Down (BeIl, RoHa, Cote) or Longwool (BlMa, Cha) genetic groups (Figs. 3 and 4). These breeds were classified either as endangered breeds (Cote, BeIl, BlMa) or local breeds with limited numbers (Char, RoHa). All these breeds (except BeIl for which there is no pedigree information) were studied by Danchin-Burge et al. [35] who considered them as having acceptable levels of genetic variability as estimated from pedigree data. More generally, for all but one (Texe) of the breeds belonging to the subdivisions *SG1* and *SG2* (hence originating from or related to British breeds, and undergoing conservation programs for six of them: Avra, Bell, Cote, Char, LaBr, RoHa), we could observe a gain of total diversity (Δ*GD*_***T***_ > 0; Table 4) if one of them was removed from the analysis. The most likely interpretation is that if one of these breeds is removed, a similar allelic combination will still exist within the set of the remaining breeds. This result illustrates quite well the fact that in a large dataset, contribution of a given breed to the total diversity depends both on its within diversity and its position within the genetic architecture of the species [31, 36, 37]. Based on our results, our recommendation would be to focus conservation efforts toward the Landaise breed (Land), although contribution to global diversity is only one of the many methods that can be used to prioritize livestock breeds for conservation. As discussed in Fabuel et al. [38] and in Leroy et al. [36], using other approaches can lead to dissimilar results. One advantage of the Caballero and Toro [33] method is that it does not suffer from the computation time limitation of the Weitzman approach for large datasets [39, 40]. It also gives the same weight to within- and between-breeds components of diversity, which can be discussed, based on what component should be emphasized for conservation purpose.

Based on these genetic diversity measures, specific recommendations can be made on the genetic management and conservation of these French breeds.

Most of the breeds that we analyzed are large breeds for which a selection program is ongoing (mainstream breeds; Table 1) and artificial insemination is used. These breeds display a wide range of genetic diversity. From this point of view, the Corsican dairy breed is clearly apart from the other breeds. As an illustration, it is the only French sheep breed with multiple color patterns since it was never submitted to coat color standardization. Lenstra et al. [41] showed that the French Corsican goat breed was more related to Italian breeds than to French breeds. The strong differentiation of the Corsican sheep breed suggests a similar history. Among this group of breeds with selection programs, the three Pyrenean dairy breeds (BaBe, MaTN and MaTR) all have also a high level of genetic diversity as well as displaying genetic distinction. These characteristics strongly support the efforts made by local organizations to promote these breeds through a PDO (protected designation of origin) product, i.e., the Ossau-Iraty cheese. Moreover, although these breeds have been subjected to fairly high selection pressures for milk production, they retain a high level of genetic diversity, probably because their genetic variability was high to start with and they have benefited from good management practices in terms of genetic variability. However, the genetic diversity of Char and the BeCh breeds is limited although the population numbers are fairly large. For both breeds, the use of artificial insemination with limited awareness of their genetic diversity combined with an erosion of the number of animals (BeCh) or a limited gene pool to start with (Char) led to a decrease in genetic diversity. Thus, we recommend that efficient measures aimed at preserving genetic diversity are taken.

Another group of breeds is composed of local breeds, which, compared to those discussed above, have a smaller population size and are part of less efficient or organized selection programs. For most of these breeds, measures of genetic diversity have intermediate values with moderate levels of inbreeding and little genetic originality. From a genetic point of view, our interpretation is that the absence or weakness of selection that is acting in these breeds preserves them. Nevertheless, for the BLMa and RoHa breeds, we recommend a short-term implementation of specific rules to slow down the rate of the loss of genetic variability.

The last group of breeds includes rare breeds. Clearly, the Land breed stands out by its high contribution to the total genetic diversity and our recommendation is to secure the existing conservation program. For instance, there are only five rams stored in the French national Cryobank (www.cryobanque.org) which is not sufficient to preserve this breed’s genetic variability in case of a disease outbreak. The same recommendation is made for the patrimonial breed, MeRa, which has a level of heterozygosity of only 0.50, which is expected for a breed that has been inbred for over 200 years.

However, regardless of our recommendations based on this work, the French Minister of Agriculture does support a global conservation of French sheep breeds. All these breeds are undergoing a selection or a conservation program, and all except MeRa are bred mostly by farmers for production. Therefore, our results rather than being used to prioritize which breeds to protect should be used as a tool to help the breeders to manage their populations.

## Conclusion

The genetic structure of French sheep breeds was shaped by reticulate evolution that involved both genetic drift and several introgression events, which correspond to similar patterns identified in Italian sheep breeds [13] or other domestic species [31, 36, 42, 43]. It is generally assumed that introgression events of Merino genetic material have occurred over the whole French sheep population [6, 7]. In comparison, introgression of UK breeds is easier to follow, probably because it is more recent. Moreover, a large part of the Merino flocks was eliminated during the second part of 19^th^ century and the beginning of the 20^th^ century [8], which resulted in a three-fold reduction of the French sheep population.

Conservation approaches could be applied to a larger number of breeds to assess conservation priorities. However, conservation issues cannot be reduced only to the analysis of genetic diversity and within- and between-population contributions. Other considerations, such as genetic structure and admixture patterns [37], or socio-cultural value and specific use of a breed, should be taken into account when making final conservation decisions [44, 45].

## Additional files

Additional file 1: Figure S1. STRUCTURE analysis with the 51 populations for *K* = 2–10 and 51. Estimated membership fractions for each individual of the 51 populations to the inferred *K* cluster, using Q-matrix averaged overall 50 runs. Additional file 2: Figure S2. STRUCTURE analysis with the 51 populations. Evolution of (a) likelihood Ln(P(D)) and (b) similarity (*G'*) according to the number of clusters *K* (*K* = 1–15, 20, 25, 30, 35, 40, 48, 50, 51, and 55).
