# Supplementary material for: How do introgression events shape the partitioning of diversity among breeds: a case study in sheep
Source: Genet Sel Evol. 2015 Jun 17;47(1):48. doi: 10.1186/s12711-015-0131-7 (PMC4470023; doi:10.1186/s12711-015-0131-7)
Supplement: Additional file 1: Figure S1. — STRUCTURE analysis with the 51 populations for K = 2–10 and 51. Estimated membership fractions for each individual of the 51 populations to the inferred K cluster, using Q-matrix averaged overall 50 runs. [file 12711_2015_131_MOESM1_ESM.pdf]

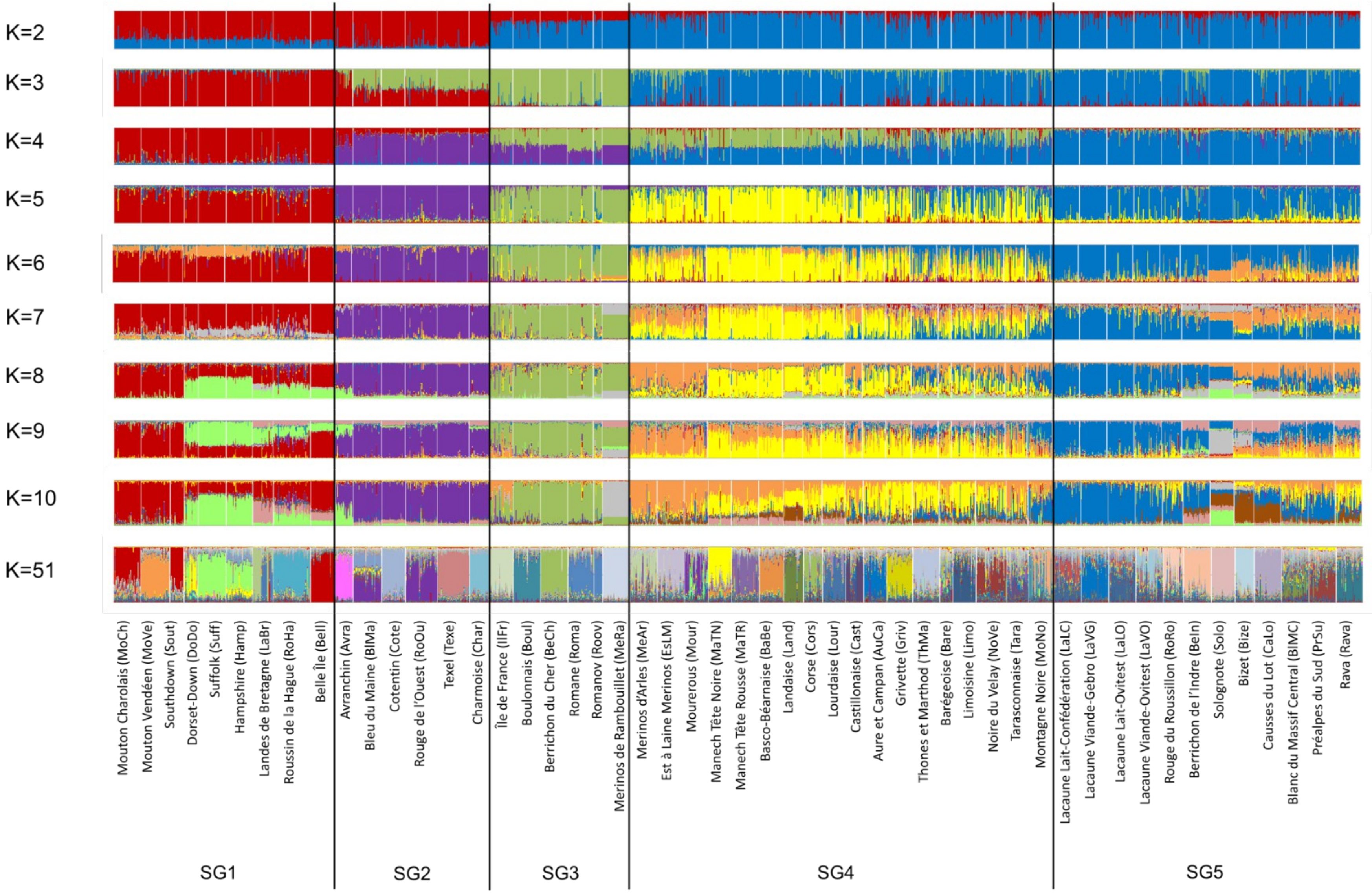

Additional file 1: Structure analysis with the 51 populations for K=2-10 and 51. Estimated membership fractions for each individual of the 51 populations to the inferred *K* cluster, using Q-matrix averaged overall 50 runs.
