# Supplementary material for: How do introgression events shape the partitioning of diversity among breeds: a case study in sheep
Source: Genet Sel Evol. 2015 Jun 17;47(1):48. doi: 10.1186/s12711-015-0131-7 (PMC4470023; doi:10.1186/s12711-015-0131-7)
Supplement: Additional file 2: Figure S2. — STRUCTURE analysis with the 51 populations. Evolution of (a) likelihood Ln(P(D)) and (b) similarity (G') according to the number of clusters K (K = 1–15, 20, 25, 30, 35, 40, 48, 50, 51, and 55). [file 12711_2015_131_MOESM2_ESM.pdf]

(a)

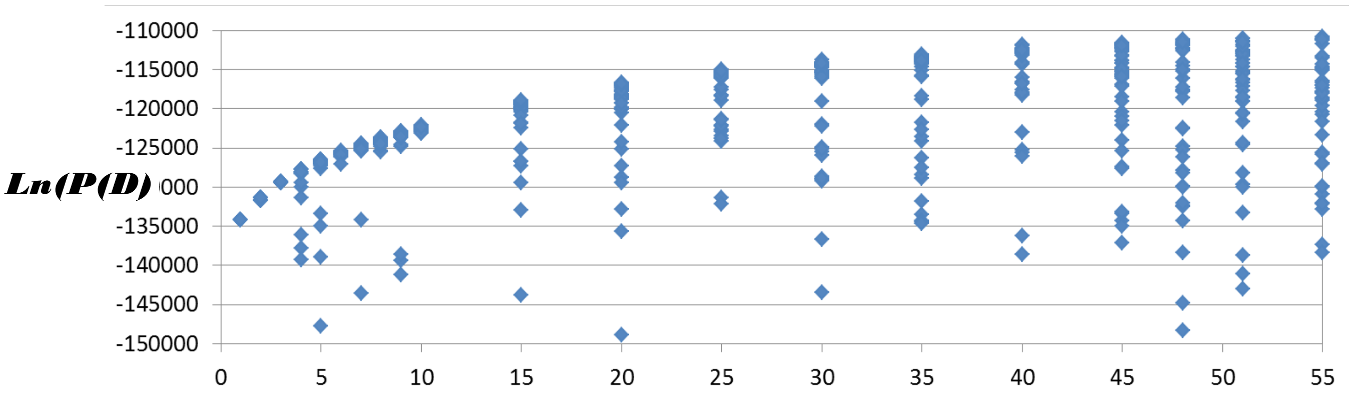

(b)

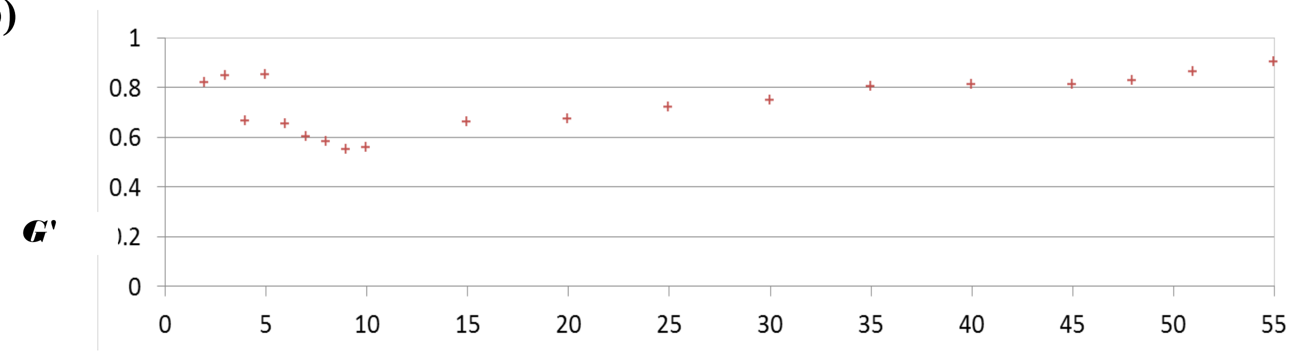

Additional file 2 : STRUCTURE analysis with the 51 populations. Evolution of (a) likelihood *Ln(P(D))* and (b) similarity (*G'*) according to the number of cluster K (K=1-10, 20, 25, 30, 35, 40, 45, 48, 51, and 55).
